# Identification of MicroRNAs in *Meloidogyne incognita* Using Deep Sequencing

**DOI:** 10.1371/journal.pone.0133491

**Published:** 2015-08-04

**Authors:** Yunsheng Wang, Zhenchuan Mao, Jin Yan, Xinyue Cheng, Feng Liu, Luo Xiao, Liangying Dai, Feng Luo, Bingyan Xie

**Affiliations:** 1 Hunan Provincial Key Laboratory for Biology and Control of Plant Diseases and Insect Pests, Hunan Agricultural University, Changsha, PR China; 2 Institute of Vegetables and Flowers, CAAS, Beijing, PR China; 3 College of Life Sciences, Beijing Normal University, Beijing, PR China; 4 School of Computing, Clemson University, Clemson, South Carolina, United States of America; James Hutton Institute, UNITED KINGDOM

## Abstract

MicroRNAs play important regulatory roles in eukaryotic lineages. In this paper, we employed deep sequencing technology to sequence and identify microRNAs in *M*. *incognita* genome, which is one of the important plant parasitic nematodes. We identified 102 *M*. *incognita* microRNA genes, which can be grouped into 71 nonredundant miRNAs based on mature sequences. Among the 71 miRANs, 27 are known miRNAs and 44 are novel miRNAs. We identified seven miRNA clusters in *M*. *incognita* genome. Four of the seven clusters, *miR-100/let-7*, *miR-71-1/miR-2a-1*, *miR-71-2/miR-2a-2* and *miR-279/miR-2b* are conserved in other species. We validated the expressions of 5 *M*. *incognita* microRNAs, including 3 known microRNAs (*miR-71*, *miR-100b* and *let-7*) and 2 novel microRNAs (*NOVEL-1* and *NOVEL-2*), using RT-PCR. We can detect all 5 microRNAs. The expression levels of four microRNAs obtained using RT-PCR were consistent with those obtained by high-throughput sequencing except for those of let-7. We also examined how *M*. *incognita* miRNAs are conserved in four other nematodes species: *C*. *elegans*, *A*. *suum*, *B*. *malayi* and *P*. *pacificus*. We found that four microRNAs, *miR-100*, *miR-92*, *miR-279* and *miR-137*, exist only in genomes of parasitic nematodes, but do not exist in the genomes of the free living nematode *C*. *elegans*. Our research created a unique resource for the research of plant parasitic nematodes. The candidate microRNAs could help elucidate the genomic structure, gene regulation, evolutionary processes, and developmental features of plant parasitic nematodes and nematode-plant interaction.

## Introduction

*Meloidogyne incognita* is a world-wide serious plant pathogen that can infect almost all cultivated plants and cause billions of dollars in losses annually [1]. Currently, the draft genomic sequences of *M*. *incognita* [2] are available, which can help elucidate the biology of RKN and their interaction with hosts.

MicroRNAs are small (~22 nt) RNAs that target the mRNAs and regulate their degradation and transcription [3, 4]. Increasing evidence has demonstrated that microRNAs play a key function in many biological processes such as tissue identity, response to environmental stress, and developmental timing [5]. MicroRNAs were first identified in *C*. *elegans* [6] and are highly evolutionarily conserved in other species. MicroRNAs are found in various eukaryotes, including plants [7, 8], animals [5, 9] and viruses [10]. It is an important step to identify microRNAs in organisms for elucidating their genome biology and evolution [11]. Although there are hundreds of microRNAs identified in different nematodes, such as *C*. *elegans* [12] and *C*. *briggsae* [13], to our knowledge, there is no report of microRNAs of plant parasitic nematodes yet. The availability of draft genomic sequences of *M*. *incognita* makes it possible to identify its microRNAs on a genome-wide level.

This research used two major approaches to identify microRNAs: (1) the direct cloning approach by cloning and sequencing the microRNAs enriched libraries [14, 15] and (2) computational prediction [16–19]. Although increasing sequences available in the public databases, including expressed sequence tags (ESTs), genome survey sequences (GSS), and high throughput genomic sequences (HTGS), made it possible to identify microRNAs by computational prediction, there are two drawbacks for computational prediction of microRNAs. First, the available nucleotide sequences in the database are limited. Computational prediction methods based on a homology search cannot predict new microRNAs if they do not exist in the database. Second, it is hard to validate the predictions using experiments because of the high false positive rate in computational prediction results. Recently, deep sequencing technology has been extensively used in microRNA genes discoveries in many species [20–27]. In this study, we employed the deep sequencing technology to sequence and identify microRNAs in the *M*. *incognita* genome.

## Methods

### Preparation of specimens

Nematode inoculums were obtained from a population of *Meloidogyne incognita* (Kofoid and White) isolated from pepper root and were reproduced in greenhouse with pepper cultivar Qiemen (*Capsicum annuum* L. cv), a RKN-susceptible cultivar. Inoculums consisted of freshly hatched juveniles from egg masses. RKN J2 were concentrated and filtered off foreign matter through a 1 mm pore size nylon sieve. Seedlings of pepper cultivars were grown in a greenhouse (25°C–28°C). The samples were collected quickly. Then, the samples were snap-frozen in liquid nitrogen and stored at -80°C.

### Small RNA libraries construction and DNA sequencing

We extracted total RNA from each RKN samples (about 1 × 10^6^ individuals) using TRIzol reagent following the manufacturer’s recommended protocol (Invitrogen, USA). The RNA quality was examined using Bioanalyzer (Agilent2100) with RIN>8.0. We collected and purified RNAs between 10–30 nt using 15% polyacrylamide gel electrophoresis (PAGE) for the sample. After PAGE purification, we added a pair of adaptors to the ends of the small RNAs according to the Illumina TruSeq Small RNA Library Prep protocol. In briefly, a 5’ adaptor (Illumina, San Diego, CA, USA) was ligated to the 5’ ends of the small RNAs and the ligation products were purified on Novex 15% PAGE. Then, a 3’ adaptor (Illumina) was ligated to the first ligation product and further purified on Novex 10% PAGE. The small RNAs were converted to cDNA by RT-PCR and then 6% TBE-Urea gel (Invitrogen) was used to purify the amplification products. Finally, the DNA fragments were used for the high-throughput sequencing. The sequencing process was done in BGI (Beijing Genome Institute at Shenzhen) using the Illumina Genome Analyzer according to the manufacturer’s instructions.

### Preprocessing of microRNAs Sequencing Data

The raw data was processed by a bioinformatics’ pipeline and include the following steps: (1) Remove low quality reads. Reads with quality score lower than 20 were removed. (2) Trim 3' prime adaptor sequences. (3) Remove adaptor-only contaminants. (4) Collect short RNAs ranging from 10 to 30 nt. Too short (<10 nt) and too long (>30 nt) reads were removed. (5) Remove sequences with polyA tails. Raw data are available at NCBI-GEO with accession number: GSE24833.

### Analysis of *M*. *incognita* microRNAs

We grouped the identical clean reads into unique sequence tags (unitags). The abundance of each unitag was indicated by the number of reads belonging to it. We used bowtie [28] to map the unitags to the draft genome of *M*. *incognita*, which was downloaded from wormbase (WS205). We only used perfectly matched reads to identify microRNA genes. We employed the miRDeep2 [29] to map sequencing tags. We only kept the candidate precursors with hairpin-like structures, which were perfectly mapped by sequencing tags. We then used the default parameters of miRDeep2 to predict the precursors and the mature sequences of microRNA genes. Finally, the candidate precursor and mature microRNAs were checked manually for secondary structure and sequenced profiles.

Then, we aligned mature microRNAs of *M*. *incognita* to known microRNAs downloaded from the miRBase database (version 21) [30] (http://www.mirbase.org/) with BlastN [31]. Those microRNAs with 80% identities and shared the same seed sequences (2–7 nt) and were supposed to be orthologous and named after the known microRNAs. If the mature sequences of two microRNA genes were identical, we treated them as the same microRNA genes with two copies in the genome, such as *miR-100a-1* and *miR-100a-2* in *M*. *incognita* with mature sequences both as uacccguagauccgaacuaguc. If the mature sequences of two microRNA genes were different from less than three bases, we labeled them as derivations of the same microRNA gene, such as *miR-100a-1* and *miR-100b*, with mature sequences such as uacccguagauccgaacuaguc and aacccguagauccgaacuagucu, respectively.

We grouped the microRNA genes into a cluster if their distances in the genome were less than 2000 bp.

### Abundance estimate of each class of small RNAs

Clean reads were aligned to each class of small RNAs sequences, including miRNAs, rRNA, tRNA, snRNA and mRNA, using bowtie [28] with default parameters except the perfect match (-v 0). Then total reads of each class were counted to estimate the abundance of expression.

### Validation of the expressions of five *M*. *incognita* microRNAs using RT-PCR

Nematodes of fresh hatched J2 were firstly exposed to freeze thaw cycles fusing liquid nitrogen and a 30°C water bath three to four times. Then the total RNAs were extracted using the TRIzol reagent (Invitrogen). The cDNA fragment was synthesized from total RNA using Superscript III reverse transcriptase (Invitrogen). The microRNA primers designed according to the premature microRNA sequences (S2 Table).

Real-time qPCR was performed on CFX96 Real-time PCR Detection System (Bio-Rad, USA) with 1.1 software, as follows: 95°C for 30 s, followed by 40 cycles of 95°C for 10 s, 58°C for 30 s, using qPCR SYBR Premix Ex Taq II for fluorophore SYBR green with fluorescein (Takara, Japan). Standard curves were established with five serial dilutions of first-strand cDNAs, ranging from 1 to 1/10 000. As reference, the *M*. *incognita* 18S ribosomal cDNA (GeneBank accession number U81578) was amplified using the primers 18S-F-852 and 18S-R-966 [32]. Relative quantity of gene expression was calculated and normalized to 18S ribosomal. The real-time qPCR were carried out with 5 technical repeats. We used the values of 2^-ΔCt^ as the gene expression abundance level [33].

### Identification and phylogenetic analysis of Argonaute protein family in *M*. *incognita*

The Argonaute protein family, which is defined by the presence of PAZ (Piwi-Argonaute-Zwille) and PIWI domains, was first identified in plants [34]. The Argonaute protein family could be phylogenetic divided into the Ago subfamily and the Piwi subfamily [35]. In general, the expression of Piwi proteins is restricted to the germ line, where they bind Piwi-interacting proteins (piRNAs). We identified the Argonaute family proteins based on the Pfam domains. Firstly, we searched the PAZ (PF02170) and Piwi (PF02171) domains in the *M*. *incognita* proteins using hmmsearch [36] against Pfam database with the e-value less than 0.01 [37]. The proteins with both PAZ and Piwi domains were identified as Argonaute family proteins. In total, we identified 15 Argonaute family proteins in the *M*. *incognita* draft genome. We also used the same method to identify Argonaute proteins from the genome of *C*. *elegans*, *B*. *malayi*, *B*. *xylophilus* and *M*. *hapla*, which were downloaded from WormBase (version WS243). We aligned Argonaute family proteins from five genomes using MAFFT [38]. Then we trimmed the alignment using trimAL [39] with parameter–automated1. Finally, we constructed a phylogenetic tree using PhyML [40] with default parameters.

## Results

### Overview of the small RNA sequencing results

**Fig 1:**
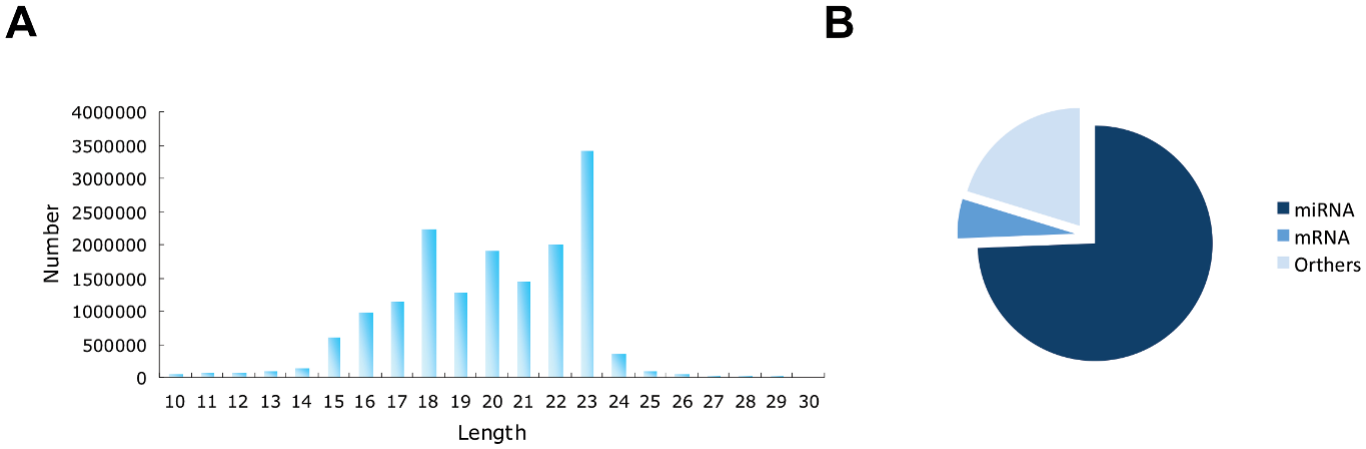
General description of the small RNA sequences of *M*. *incognita*. (A) Size distribution of the raw reads of *M*. *incognita* small RNAs. (B) Classification of the small RNA reads.

We obtained 18,509,803 raw reads from the small RNA library of J2 juveniles of *M*. *incognita*. After removing low-quality bases, contaminants and masking adaptor sequences, we obtained 16,020,648 clean reads. The clean reads were mainly distributed between 15 and 23 nt (15,005,173, 93.7%) and had a peak length of 23 nt (Fig 1A). We were able to group the clean reads into 761,538 unique tags based on their sequence similarity. The most abundance sequence tag had 1,925,637 reads. 90.6% of the clean reads (14,515,814) can be mapped onto the draft genome sequences of *M*. *incognita* using bowtie [28] with no mismatches (-v 0).

**Fig 2:**
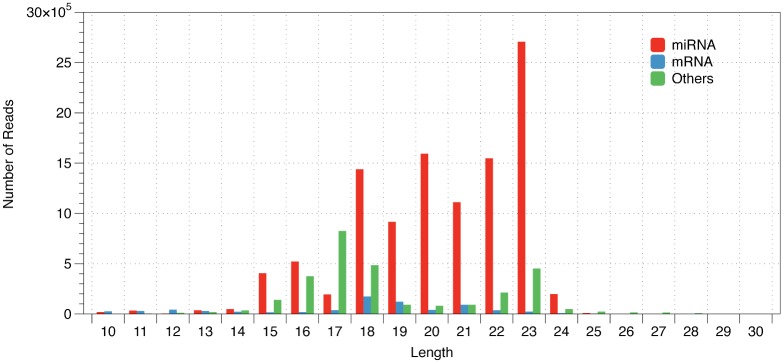
Size distribution of reads from microRNA, protein encoding genes and others.

To annotate the small RNAs, we aligned the clean reads against the microRNAs, tRNA, rRNA, snRNA and mRNA sequences of *M*. *incognita* and then counted the reads of each class. There were 74.35%, 5.38% and 1.10% of clean reads mapped to predicted microRNA, protein encoding and tRNA genes, respectively. There were 19.14% of clean reads mapped to other classes, including rRNA, snRNA, and siRNA (Fig 1B). There were 9.4% of clean reads that were unable to be mapped to the *M*. *incognita* genome with no mismatches. The top 10 most abundance sequence tags were all microRNAs. The lengths of the microRNA reads were mainly distributed between 18 and 23 nt, which include 86.34% of the total microRNA reads (Fig 2).

### Identification and validation of microRNA genes of *M*. *incognita*

**Fig 3:**
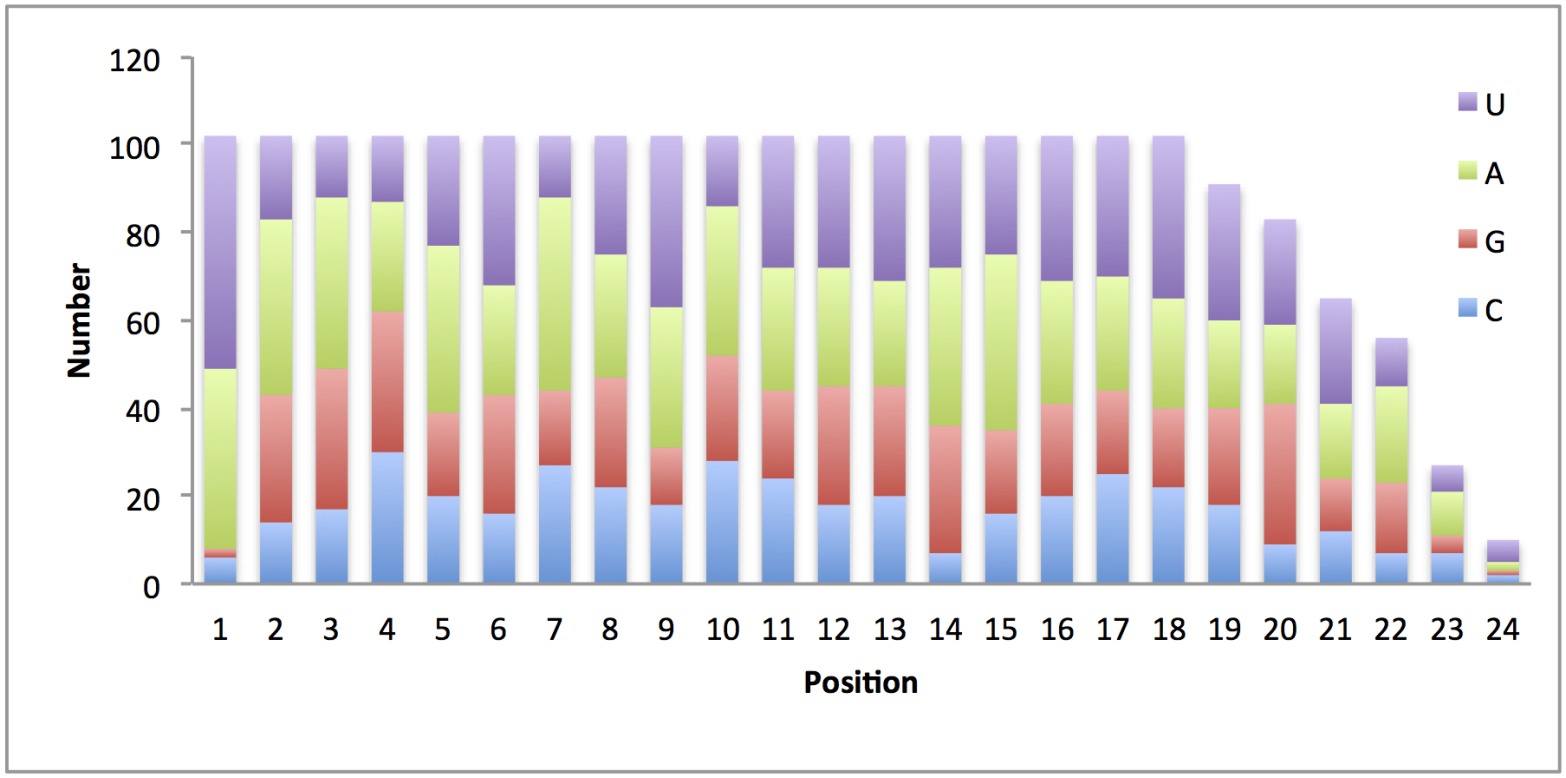
Distribution of bases at each position of mature microRNAs. The first base of mature microRNA tend to be A and U.

**Fig 4:**
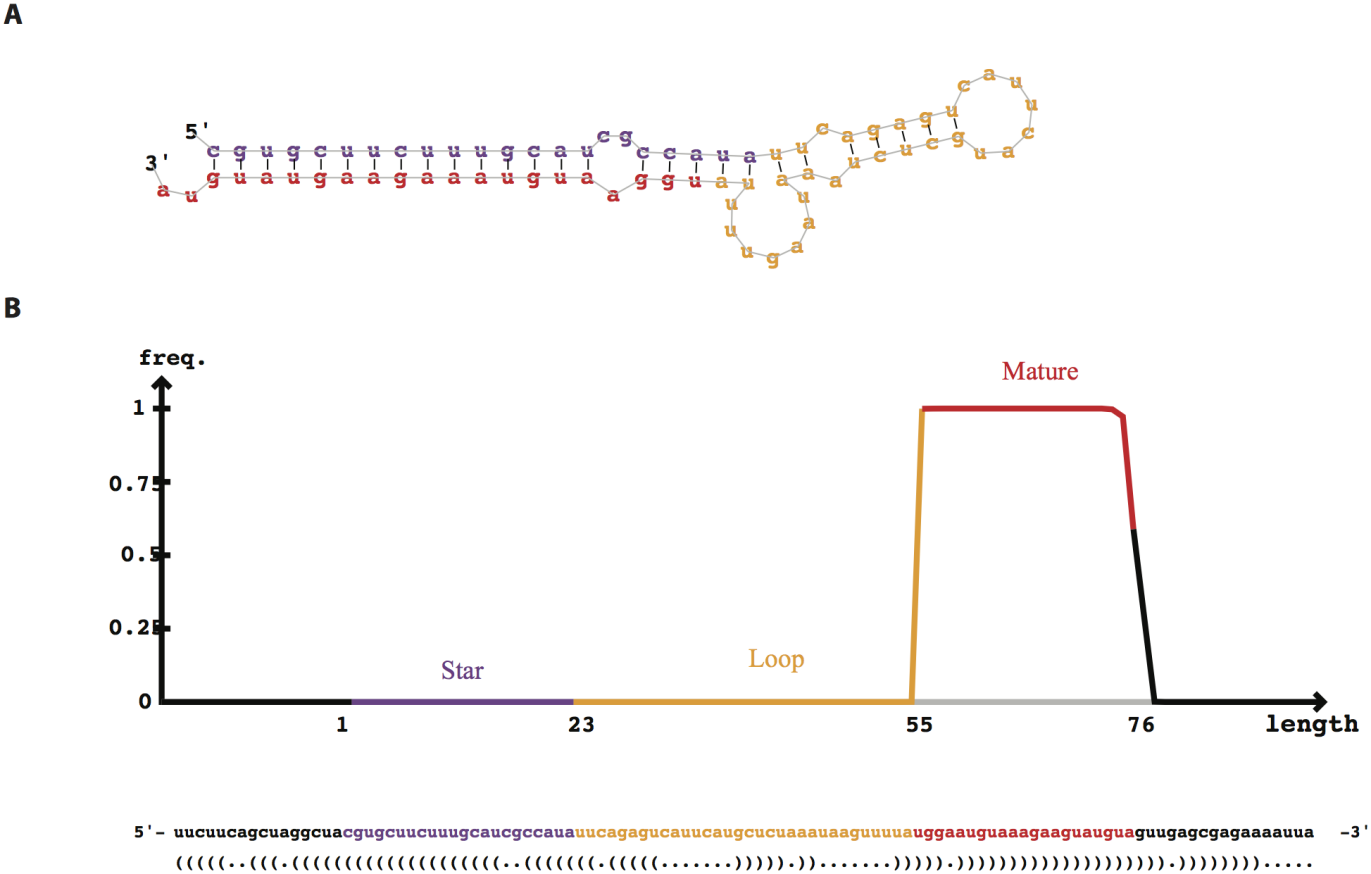
*min-miR-1* hairpin structure and sequencing profile. (A) Hairpin structure predicted with RNAfold. (B) Proportion of small RNA tags mapped to the pre-miRNA of *min-miR-1*.

We identified 102 candidate microRNA genes of *M*. *incognita* (S1 Table) using miRDeep2 with a score of more than 0. We then predicted the mature microRNA from precursor microRNAs. The mature microRNAs were from 18 to 24 nt in length. We noted a significant bias of A and U at the first position of mature microRNAs (Fig 3B). 94 out of 102 mature microRNAs started with A or U (41 microRNAs started with A, 53 microRNAs started with U). The other parts of precursor microRNAs besides mature microRNAs usually undergo the degradation process. As a result, the mature microRNA had a significantly deeper coverage than those of the other parts of microRNA genes, such as the star microRNA (miRNA*), the other strand of mature microRNA on the hairpin structure of precursor microRNA, and loop sequences (the sequences between mature miRNA and miRNA*). Most of the putative pre-miRNAs had a very high read depth in mature arm and a much low depth in the other arm with a typical hairpin structure (Fig 4).

Based on mature microRNA sequence, we were able to group 102 microRNAs into 71 unique microRNA genes. Among them, 25 microRNA genes had multiple copies in the draft genome of *M*. *incognita*. From those 71 unique miRNAs, we identified 27 known miRNA families, which are known in the miRBase database (Table 1).

**Table 1:** Known miRNA families identified in *M*. *incognita*.

Name	Mature	Length	GC%	Reads number
*miR-71*	ugaaagacauggguaguugaga	22	40.9%	3489388
*miR-100b*	aacccguagauccgaacuagucu	23	47.8%	2870101
*miR-124*	uaaggcacgcggugaaug	18	55.6%	1639977
*miR-1*	uggaauguaaagaaguau	18	27.8%	880854
*miR-72*	aggcaagauguuggcauugcuga	23	47.8%	495031
*miR-92*	uauugcacucguuucggccu	20	50.0%	313210
*miR-252*	cuaaguaguagugccgcauuuaa	23	39.1%	65196
*miR-2a*	uaucacagccugcuuuagcgua	22	45.5%	57375
*miR-87*	gugagcaaaguuucaggugugc	22	50.0%	42872
*miR-100a*	uacccguagauccgaacuaguc	22	50.0%	28784
*miR-2b*	uaucacaguucgauauggcc	20	45.0%	25154
*miR-50*	ugauaugucuuguauucuug	20	30.0%	20539
*miR-184*	uggacggaagucugauaaggag	22	50.0%	17866
*miR-81*	ugagaucauaccagaucac	19	42.1%	15488
*miR-86*	uaagugaauaucuugccacaagcu	24	37.5%	9234
*miR-279*	ugacuagauccacacucaucu	21	42.9%	9908
*miR-137*	agguauucuccguggugaugaca	23	47.8%	6368
*miR-59*	acgaaucguuugcacaucgguguu	24	45.8%	6103
*miR-79*	auaaagcuagauuaccagag	20	35.0%	5635
*miR-67*	ucacaacccccuagaguucgcua	23	52.2%	5292
*miR-239*	uuuguacuagccaaaaugucugca	24	37.5%	4576
*miR-36*	ucaccgggaauuuauucaug	20	40.0%	698
*let-7*	ugagguaguagguuguauaguu	22	36.4%	223
*miR-242*	uugcguaggcaucuugucag	20	50.0%	121
*miR-240*	cacuggccuuucaaaccu	18	50.0%	60
*miR-76*	uucguuguuucugaaaccugaa	22	36.4%	12
*miR-790*	acgguuugacaaaguuau	18	33.3%	9

**Fig 5:**
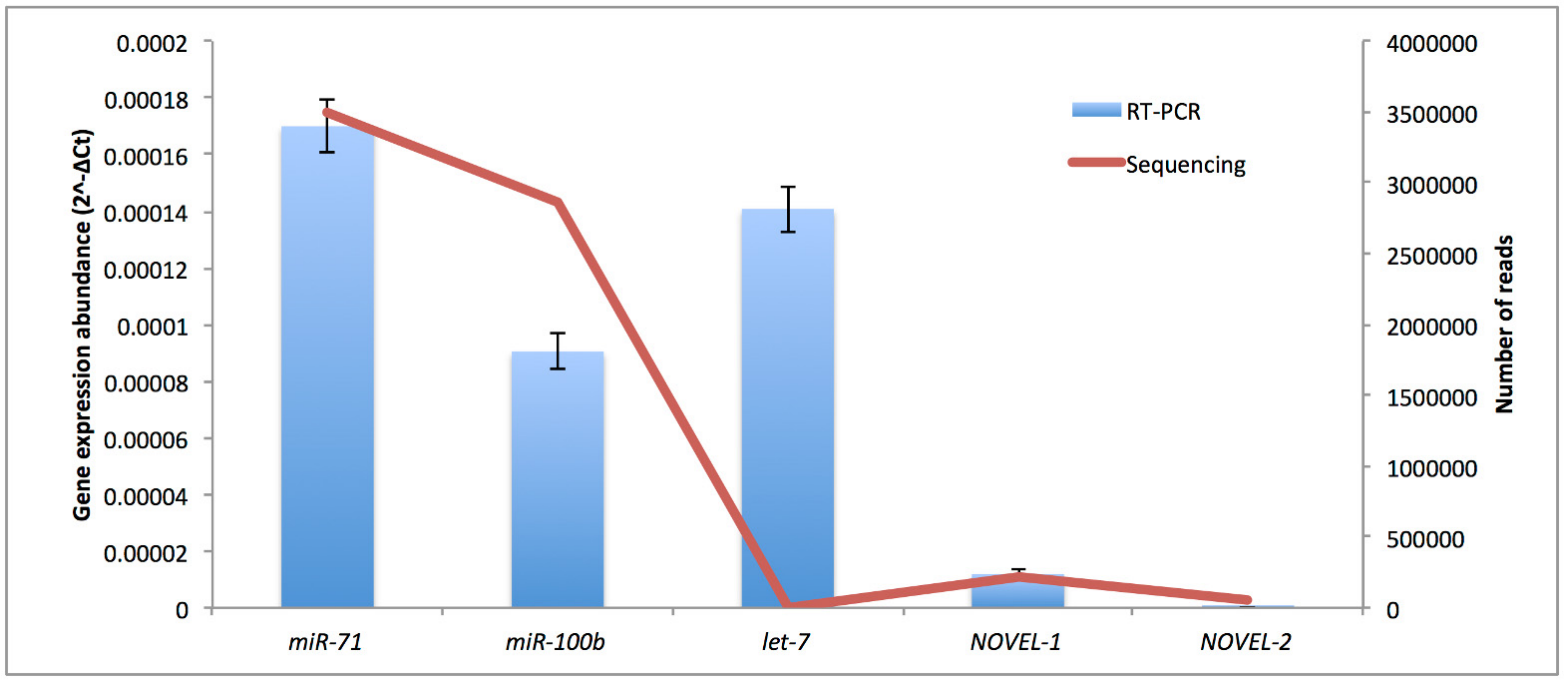
The expression abundance of microRNAs detected by real time RT-PCR (bars) and by high-throughput sequencing (lines).

We selected 5 microRNAs for validation using RT-PCR, including 3 known microRNAs and 2 novel microRNAs of *M*. *incognita*. All of the 5 microRNAs could be detected using real time RT-PCR with the mature microRNAs as a primer. The microRNA expression was determined using real time RT-PCR by 2^-ΔCt^ measurements. The expression levels of four microRNAs, *miR-71*, *miR-100b*, *NOVEL-1* and *NOVEL-2*, were consistent with those obtained by high-throughput sequencing. The expression levels of *miR-71* and *miR-100b* were much higher than those of *NOVEL-1* and *NOVEL-2* in both results of sequencing and qRT-PCR. However, the expression abundance of let-7 detected by real time RT-PCR is much higher than that by high-throughput sequencing (Fig 5).

### Identification of microRNA clusters in *M*. *incognita*

MicroRNAs are often clustered in the genome [30]. We identified seven microRNA clusters in *M*. *incognita* genome (Table 2), and four of them were also found in other species.

**Table 2:** List of the miRNA clusters of *M*. *incognita*.

Contig	Position	Strand	Cluster	Reported in other nematode
MiV1ctg20	43319–43619	+	*miR-71-1*	*H*. *contortus* [48]
			*miR-2a-1*	
MiV1ctg221	69290–69588	-	*miR-71-2*	
			*miR-2a-2*	
MiV1ctg1924	3647–4158	+	*let-7*	*B*. *malayi* [41]
			*miR-100*	
MiV1ctg644	18822–19030	-	*miR-279*	*B*. *pahangi* [48]
			*miR-2b*	
MiV1ctg27	99326–99923	-	*NOVEL-1-1*	
			*NOVEL-39*	
MiV1ctg2865	1792–2014	+	*miR-240*	
			*NOVEL-30*	
MiV1ctg1143	4187–4580	+	*NOVEL-12*	
			*NOVEL-11*	
			*NOVEL-14*	

**Fig 6:**
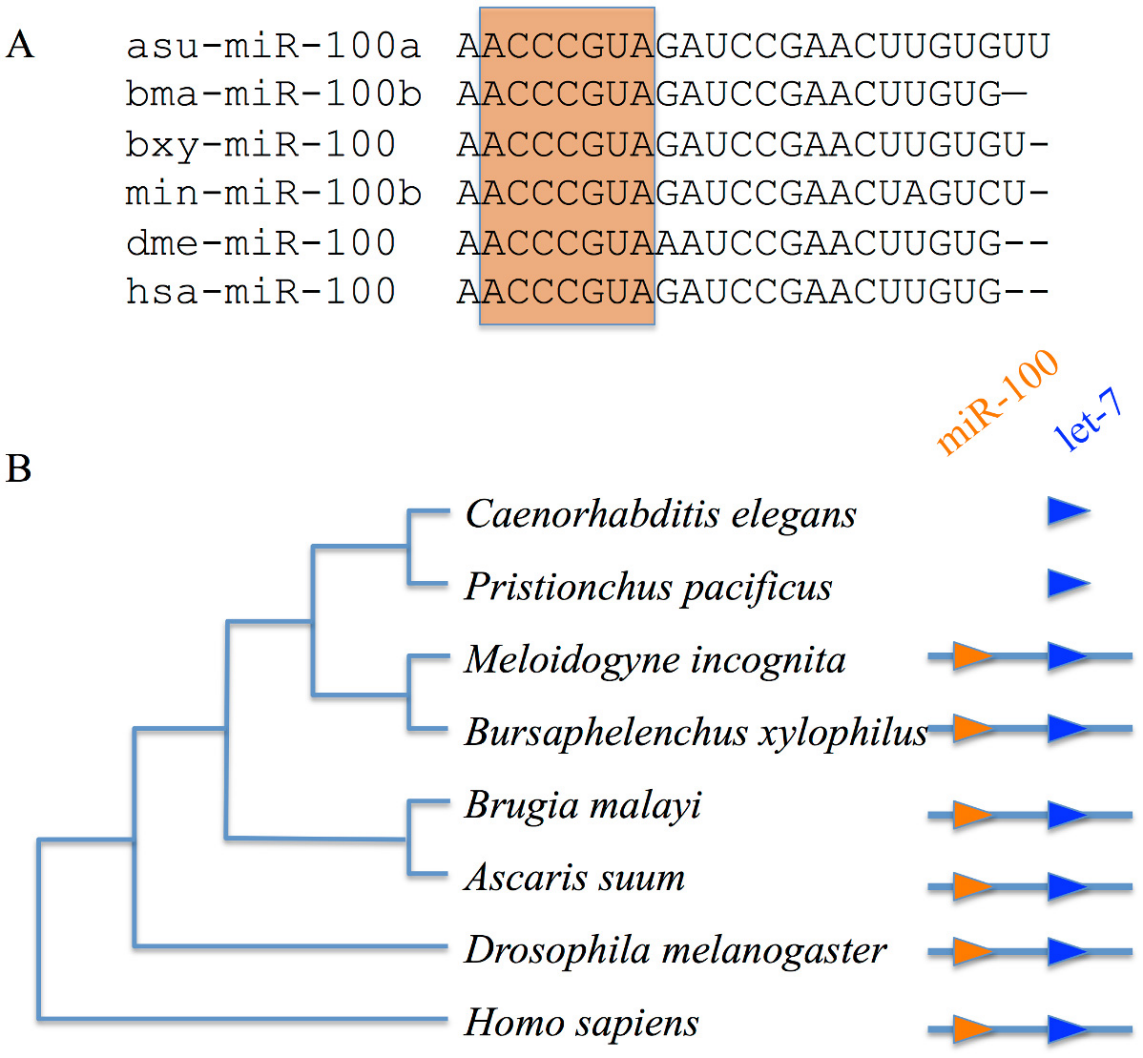
Conservation of *miR-100*, in sequence and genomic organization. (A) Sequence alignment of *miR-100* microRNAs among different species (asu-: *A*. *suum*, bma-: *B*. *malayi*, bxy-: *B*. *xylophilus*, min-: *M*. *incognita*, dme-: *D*. *melanogaster*, has-: *H*. *sapiens*). The seed sequences indicated with red square area. (B) *miR-100* and *let-7* were clustered together in diverse animals but the *miR-100* had lost in the common ancestor of *C*. *elegans* and *P*. *pacificus*.

*MiR-100* orthologues are often found in clusters with *let-7* and the clusters range in size from ~300 to 4000 bp [41]. The microRNA cluster *let-7* and *miR-100* has been found in *Brugia malayi* [41], *Drosophila* [42] and humans [43]. In the *M*. *incognita* genome, the *miR-100* is clustered within ~350 bp of *let-7*. Many organisms express multiple *miR-100* paralogues. There are four paralogues (*miR-100a* through 100d) in *B*. *malayi*. We have identified 3 *miR-100* paralogues in *M*. *incognita*. The sequences alignment of the *miR-100* orthologues from human, fly, *B*. *malayi*, *A*. *suum*, *B*. *xylophilus*, and *M*. *incognita* showed that the seed sequence, ACCCGUA, conserved in these species (Fig 6A). It is interesting that *miR-100* has been lost in free living nematodes such as *C*. *elegans* [44] and *P*. *pacificus* (Fig 6B).

The *miR-71*/*miR-2* cluster is found in two locations in *M*. *incognita*. Previous functional analysis showed that *Drosophila miR-2* is associated with the suppression of embryonic apoptosis [45]. The *miR-71* of *C*. *elegans* is related to lifespan, stress response [46]. The *miR-71* of *C*. *elegans* also was reported to function in neurons to promote germline-mediated longevity and facilitates the localization and activity of DAF-16 in the intestine [47]. The *miR-71*/*miR-2* cluster was also found in *H*. *contortus* [48], which also suggest the functional linkage of these two miRNAs.

The *miR-279* and *miR-2b* were also in a close cluster in *M*. *incognita*. The *miR-279* was reported to regulate the JAK/STAT pathway to drive rest:activity rhythms in *Drosophila* [49].

### The highly expressed microRNA genes in *M*. *incognita*

**Fig 7:**
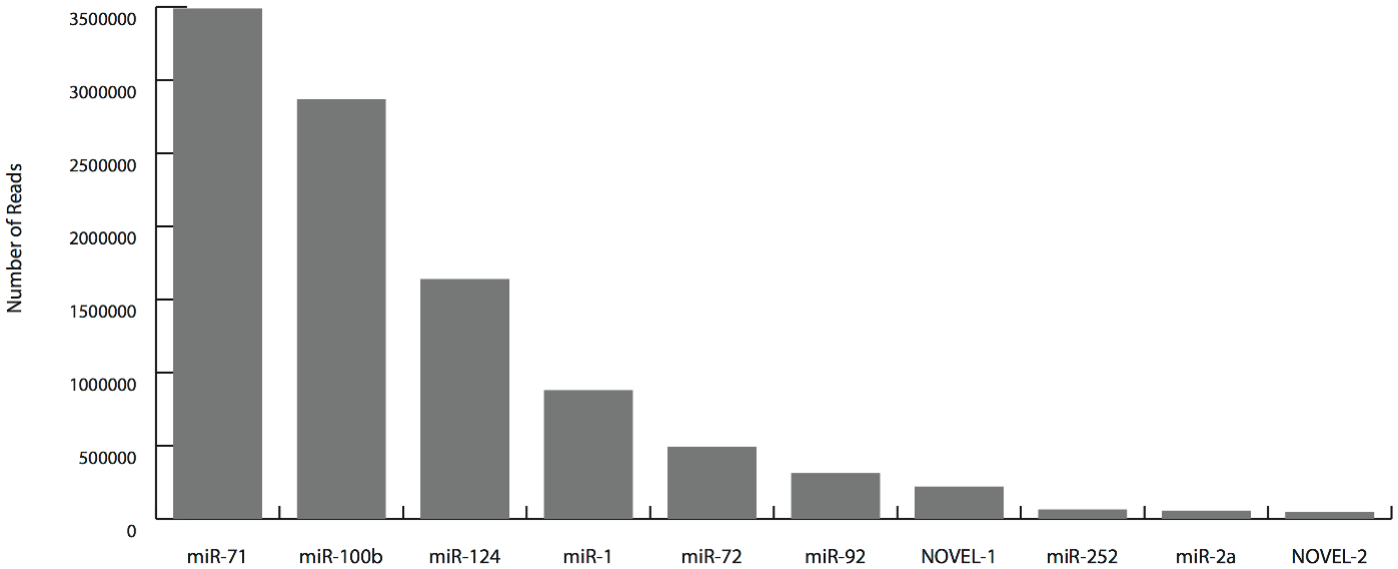
The top 10 abundance miRNAs in *M*. *incognita* J2 library

Few microRNA genes are highly expressed in our sequencing data. The numbers of reads for the top 10 abundance microRNAs are shown in Fig 7. The first two abundance microRNAs, *miR-71* and *miR-100b*, have 6,359,489 reads, which are approximately 50% of the total clean reads. The very high expression level indicated that these miRNAs may be important to the life of *M*. *incognita*. Interestingly, there are two microRNAs, *miR-100* and *miR-92*, that were highly expressed in *M*. *incognita*, but were lost in *C*. *elegans*.

The most expressed microRNA, *miR-71*, has important roles in extending the life span in *C*. *elegans* after germline removal [47]. The *miR-71* regulates the DAF-16/FOXO in neurons to enhance germline-mediated longevity [47]. It has also been reported that *miR-71* can target the TIR-1/Sarm1 adaptor protein to inhibit calcium signaling pathway [50].

The second most expressed microRNA, *miR-100*, was found to be an oncogene in human, which is differently expressed in many cancer cells [51]. In nasopharyngeal cancer, *miR-100* regulates the expression of Polo-like kinase 1 [52]. In adrenocortical cancer cells and in clear cell ovarian cancer, *miR-100* targets mTOR [53]. In acute myeloblastic leukemia, *miR-100* targets the RBSP3 to regulate cell differentiation and survival [51].

The third highly expressed microRNA, *miR-124*, may function in the neural cell. The human *miR-124* is the most abundant microRNA expressed in neuronal cells although the differentiation was not affected by the changing of *miR-124* expression in neural cells [54]. The mice *miR-124* regulated the temporal progression of adult neurogenesis. Suppressing *miR-124* function during regeneration caused hyperplasias and neurogenesis delay in mice [55].

The fourth abundant microRAN, *miR-1*, is a muscle-specific microRNA. The *miR-1* controls both pre- and postsynaptic function in *C*. *elegans* neuromuscular junctions [56]. The *miR-92* gene has been found in *B*. *malayi* and *A*. *suum*. It is also a key oncogenic gene in colon cancer in humans [57].

### Lack of piRNA and piRNA pathway components in *M*. *incognita*

piRNAs are critical microRNAs for germ line cell development in many species. The generation of piRNAs employed a distinct mechanism that does not involve Dicer [58]. In *C*. *elegans*, piRNA orthologs are only 21 nt in length with a 5' terminal U, which are known as 21U-RNAs. The piRNAs are not conserved at the sequence level among other *Caenorhabditis* species and do not have significant complementarity to targets [20]. Piwi proteins and piRNAs have been found in worms, flies, sponges, and humans [59]. PiRNAs interact with Piwi proteins to form RNA-protein complexes. The Piwi Argonaute ortholog PRG-1 is required for interaction with piRNAs [60]. The piRNA-Piwi protein complex has been reported to play an important role in silencing the retrotransposons in germ line cells through regulating both epigenetic and post-transcriptional pathways, particularly those in spermatogenesis [61].

**Fig 8:**
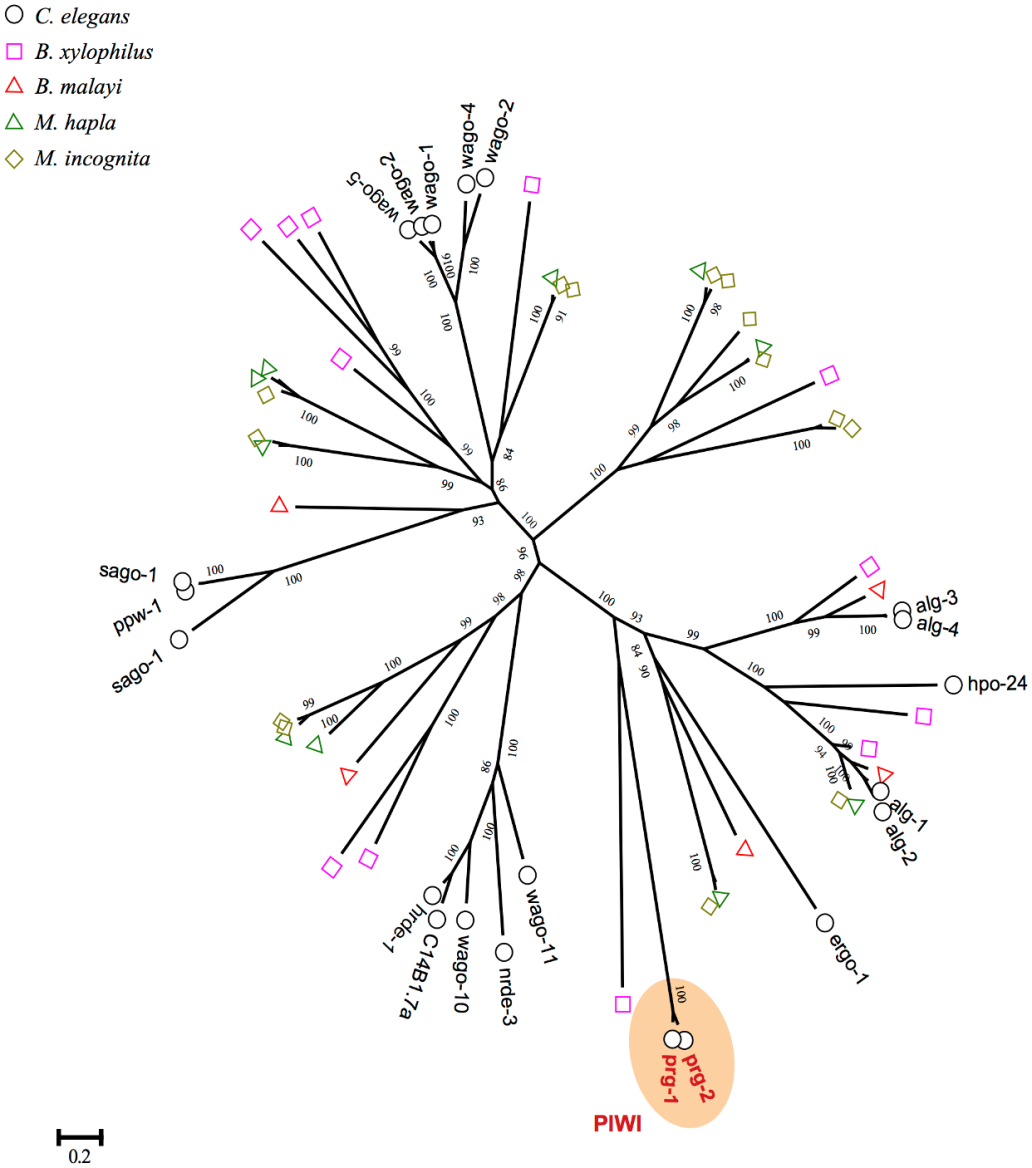
*M*. *incognita* lacks Piwi-clade argonaute proteins, which is essential for Pi-RNA biosynthesis. All of argonaute proteins, containing Piwi and PAZ domain, were identified from the genome of *C*. *elegans*, *B*. *xylophilus*, *B*. *malayi*, *M*. *hapla* and *M*. *incognita* and the multiple sequences alignment were carried out using mafft with default parameters. The phylogenetic tree was constructed using PhyML and the number on the branch indicated the bootstrap values.

However, in our small RNA sequencing results of the J2 library for *M*. *incognita*, we did not found any small RNAs with characteristics of piRNAs. Moreover, the Piwi-clade Argonaute orthologs could not be found in *M*. *incognita* genome (Fig 8). Recently, the HEN1 ortholog henn-1 was identified and proved to be required in piRNAs pathway in *C*. *elegans* [62]. However, we failed to detect any ortholog of the HEN1 methyltransferase in *M*. *incognita* genome. Notably, the Piwi-clade Argonaute and HEN1 orthologs are also not found in the parasite nematode *B*. *malayi* [63] and *A*. *suum* [64]. It was hypothesized that the piRNA pathway may be lost in *A*. *suum* [64]. In a very recent study, it is also indicated that piRNAs exist only in nematode *C*. *elegans* and closely related nematodes, and absent in all other nematode lineages [65]. The lacking of piRNAs and piRNA pathway components imply that the piRNA pathway may also be lost in *M*. *incognita*.

### Conservation of miRNAs of *M*. *incognita* in other nematodes

We examined how *M*. *incognita* miRNAs were conserved in other four nematodes species: *C*. *elegans*, *A*. *suum*, *B*. *malayi* and *P*. *pacificus*. Table 3 shows how *M*. *incognita* microRNAs exist at least in one other nematodes genome. There are 26 *M*. *incognita* microRNAs conserved in at least one other nematode genome. However, only seven *M*. *incognita* microRNAs are conserved in all five nematodes: *let-7*, *miR124*, *miR-2*, *miR-71*, *miR-72*, *miR-79*, *miR-87*. There are four microRNAs, *miR-100*, *miR-92*, *miR-279* and *miR-137*, which exist only in genomes of parasitic nematodes *A*. *suum*, *B*. *malayi*, *P*. *pacificus* and *M*. *incognita*, but do not exist in the genomes of the free living nematode *C*. *elegans*. These four microRNAs may have an important function in the parasite process.

**Table 3:** miRNAs of *M*. *incognita* conserved in other nematodes.

mir	min	cel	asu	bma	ppc
*let-7*	*	*	*	*	*
*miR-100*	*		*	*	
*miR-124*	*	*	*	*	*
*miR-137*	*		*	*	
*miR-184*	*		*		
*miR-1*	*	*	*		*
*miR-239*	*	*			*
*miR-240*	*	*			*
*miR-242*	*	*			*
*miR-252*	*	*	*		*
*miR-279*	*		*	*	*
*miR-2*	*	*	*	*	*
*miR-36*	*	*	*	*	
*miR-49*	*	*	*		
*miR-50*	*	*	*	*	
*miR-59*	*	*			
*miR-67*	*	*	*		*
*miR-71*	*	*	*	*	*
*miR-72*	*	*	*	*	*
*miR-76*	*	*	*		
*miR-790*	*	*			
*miR-79*	*	*	*	*	*
*miR-81*	*	*	*		*
*miR-86*	*	*	*		*
*miR-87*	*	*	*	*	*
*miR-92*	*		*	*	

Note: Star (*) indicates that the specific miRNAs family has been found in this species. min: *M*. *incognita*, cel: *C*. *elegans*, asu: *A*. *suum*, bma: *B*. *malayi*, ppc: *P*. *pacificus*

## Discussion and Conclusions

In this study, we generated about 18 million raw microRNA reads. After preprocessing, we eventually obtained a total of about 0.5 million non-redundant unitags with high quality reads. However, only 43.44% (232307 out of 534834) of these non-redundant small RNA unitags have a perfect match in the draft *M*. *incognita* genome. This could due to the following reasons: (1) Genetic polymorphisms: It is well known that the genetic variation of *M*. *incognita* is due to the heteroploid phenomenon; (2) Incompleteness of the genome: The public-released draft genome of *M*. *incognita* was supposed to be only part of the genome [2]. There are lots of gaps in the assembled genome; (3) Systemic errors: Sequencing errors can block perfect alignment.

In summary, this first report of microRNAs of plant parasitic nematodes and the genome-wide identification of *M*. *incognita* microRNAs have created a unique resource for the research of plant parasitic nematode. The candidate microRNAs will help researchers better understand and refine their approach to studies on genomic structure, gene regulation, evolutionary processes, and developmental features of plant parasitic nematodes and nematode-plant interaction. However, further biological experiments are needed to verify the functionalities of *M*. *incognita* microRNAs and how they regulate their target genes in the developing process.

## Supporting Information

S1 Table The microRNAs of *M*. *incognita* identified from the deep sequences. (XLSX)

S2 Table The primer sequences used in the qRT-PCR validation. (DOCX)
