# Supplementary material for: Identification of MicroRNAs in Meloidogyne incognita Using Deep Sequencing
Source: PLoS One. 2015 Aug 4;10(8):e0133491. doi: 10.1371/journal.pone.0133491 (PMC4524723; doi:10.1371/journal.pone.0133491)
Supplement: S2 Table — (DOCX) [file pone.0133491.s002.docx]

Table S2. Primer sequences used in qRT-PCR validation

| Name | Primer sequence |
| --- | --- |
| let7-F | GTTAAAGAACAGTATCAGTCGG |
| let7-R | AGTGAGCAGGCTGCGTTA |
| miR71-F | ATGGGTAGTTGAGACGTGTTG |
| miR71-R | TTGAGTATAATTCGGATGGC |
| miR100-F | CTAGTCTCTCGTGTCCGTCC |
| miR100-R | AACGCCGAGCCAGTCTA |
| Nov1-F | TCTGACTGTTATCTGCATGTTT |
| Nov1-R | GAATCTGGACTGATCTCATGAA |
| Nov2-F | TTCTTTGTGCTGCTTGGC |
| Nov2-R | TGGGCAGCTTGGACATATA |
| 18S-qF | ACCGTGGCCAGACAAACTAC |
| 18S-qR | GATCGCTAGTTGGCATCGTT |
